# Temporal Trends in Characteristics of Newly Diagnosed Nontraumatic Osteonecrosis of the Femoral Head From 1997 to 2011: A Hospital-Based Sentinel Monitoring System in Japan

**DOI:** 10.2188/jea.JE20140162

**Published:** 2015-06-05

**Authors:** Shinji Takahashi, Wakaba Fukushima, Takuaki Yamamoto, Yukihide Iwamoto, Toshikazu Kubo, Nobuhiko Sugano, Yoshio Hirota

**Affiliations:** 1 Department of Public Health, Osaka City University Faculty of Medicine, Osaka, Japan; 2 Department of Orthopaedic Surgery, Osaka City University Faculty of Medicine, Osaka, Japan; 3 Department of Orthopaedic Surgery, Kyushu University, Fukuoka, Japan; 4 Department of Orthopaedics, Graduate School of Medical Science, Kyoto Prefectural University of Medicine, Kyoto, Japan; 5 Department of Orthopaedic Medical Engineering, Osaka University Graduate School of Medicine, Suita, Osaka, Japan

**Keywords:** nontraumatic osteonecrosis of the femoral head, temporal trends, a multicenter hospital-based sentinel monitoring system

## Abstract

**Background:**

Nontraumatic osteonecrosis of the femoral head (ONFH) is a rare disorder caused by ischemic necrosis of unknown etiology. A few studies have demonstrated trends in the number of patients with ONFH. However, there are no data on temporal trends in characteristics such as age, gender, and causative factors. To investigate this, we examined data from a multicenter hospital-based sentinel monitoring system in Japan.

**Methods:**

A total of 3041 newly-diagnosed ONFH patients from 34 participating hospitals who were reported to the system from 1997–2011 were analyzed. We examined age at diagnosis, potential causative factors, and underlying diseases for which patients received systemic steroid administration. Their temporal trends were assessed according to date of diagnosis in 5-year intervals (1997–2001, 2002–2006, and 2007–2011).

**Results:**

The gender ratio and distribution of potential causative factors did not change. Regarding underlying diseases requiring steroid administration, the proportion of patients with systemic lupus erythematosus decreased in males (10% to 6.4%) and in females (37% to 29%). Proportion of patients with renal transplantation fell consistently across the study period in both males (3.8% to 1.2%) and females (3.2% to 0.8%). In contrast, the proportion of patients receiving steroids for pulmonary disease (except asthma) significantly increased in both males (0.5% to 5.5%) and females (0.5% to 3.6%).

**Conclusions:**

This large descriptive study is the first to investigate temporal trends in the characteristics of ONFH, which provide useful information for future studies.

## INTRODUCTION

Nontraumatic osteonecrosis of the femoral head (ONFH) is a disorder of unknown pathogenesis that often progresses to hip joint destruction and physical disability.^1^ Once the destruction occurs, surgical interventions, such as osteotomy and hip replacement, are required. ONFH is a rare disease, and its annual incidence in Japan has been reported to be an average of 2.51 cases per 100 000 persons between 1999 and 2008.^2^ Some epidemiologic studies have shown an increase in the number of patients with ONFH in recent years.^3^^–^^5^ Kang et al^3^ conducted a nationwide survey between 2002 and 2006 using medical claims data from the National Health Insurance Corporation to estimate the prevalence in Korea. They reported that the prevalence increased from 20.53 per 100 000 persons in 2002 to 37.96 in 2006 (a 1.8-fold increase). Another survey from Japan reported that the estimated number of patients with ONFH who sought medical care in 2004 was 1.5 times higher than that in 1994.^4^^,^^5^ However, even for well-known risk factors, such as corticosteroid use and alcohol intake,^6^^–^^9^ the temporal trends in the characteristics of ONFH have not been investigated.

In the present study, using data collected in a multicenter hospital-based sentinel monitoring system in Japan over a period of 15 years, we evaluated temporal trends in ONFH with respect to basic characteristics, including gender ratio, age at diagnosis, potential causative factors, and underlying diseases treated by systemic steroid administration. The trends were examined according to gender because the characteristics of ONFH differ considerably between male and female patients.^5^

## METHODS

Since 1972, the Ministry of Health, Labour and Welfare in Japan has carried out a special program against so-called “intractable diseases,” which are defined as rare diseases without any established therapy. The program includes eliminating patients’ copayments for medical expenditures and promoting research activities. The Research Committee on Idiopathic Osteonecrosis of the Femoral Head, which consists of hip surgeons and experts in epidemiology, biology, and genetics from all parts of Japan, was established in 1975 under the program. The research committee started a multicenter hospital-based sentinel monitoring system for ONFH (hereafter referred to as the monitoring system) in June 1997 to elucidate the descriptive epidemiology of ONFH. The monitoring system is ongoing, and a total of 34 hospitals, including 31 university hospitals and three highly specialized medical centers, have participated up to November 2012.

When a patient was newly diagnosed with ONFH at one of the participating hospitals, the demographic and clinical information of the patient was reported to the monitoring system. Patients were also reported if they had been diagnosed in previous hospitals and then referred to the participating hospitals. The diagnosis of ONFH was made by hip surgeons based on the criteria proposed by the research committee.^10^ The diagnostic criteria had a sensitivity of 91% and a specificity of 99% when histologic diagnosis was used as the gold standard.^10^ Patients with caisson disease or trauma history of the hip joint were excluded because of secondary osteonecrosis due to external factors. The study protocol was approved by the ethics committees of each participating hospital.

### Data collection

A structured form was used to collect patients’ information on demographic and clinical characteristics. The form included the following as basic information: date of birth, gender, date of disease diagnosis, potential causative factors, and any underlying disease for which patients received systemic steroid administration. Assessment of potential causative factors comprised four categories, which were a combination of two major risk factors for ONFH: history of systemic steroid administration, history of habitual alcohol intake, history of both, and history of neither. All of the underlying diseases for which steroids were administered were reported. We did not use any specific definition of habitual alcohol intake because there is no universal criterion of alcohol-induced ONFH. As additional information, data on the amount and duration of alcohol intake among ONFH patients was available for those who were reported to the monitoring system after 2009. A representative hip surgeon in each participating hospital was asked to complete or recheck the data if the information was missing or lacked consistency.

### Data analysis

All analyses were performed based on the calendar year of diagnosis. Among the patients reported up to November 2012, those who were first diagnosed with ONFH between January 1997 and December 2011 (a total of 15 years) were extracted for the present study. The date of diagnosis was further divided into 5-year intervals, resulting in three periods to assess trends in the characteristics: the first period, 1997–2001; the second period, 2002–2006; and the third period, 2007–2011. We further excluded the following patients: patients with missing data on gender or age; patients who were reported to the monitoring system more than 3 years after diagnosis, because a longer period since diagnosis could introduce a reverse relationship between causative factors or underlying disease treated with steroids and development of ONFH; and patients aged 15 years or less, to avoid the possible inclusion of Perthes disease.

Underlying diseases treated by steroid administration were categorized as follows: systemic lupus erythematosus (SLE), rheumatoid arthritis (RA), polymyositis/dermatomyositis, mixed connective tissue disease, Sjögren syndrome, other types of collagen disease, nephrotic syndrome, nephritis, renal transplantation, other organ transplantation (except renal transplantation and bone marrow transplantation), hematological malignancy, thrombocytopenic purpura, aplastic anemia, inflammatory bowel disease, hepatitis, bronchial asthma, pulmonary disease (except asthma), skin disease, eye disease, ear disease, facial palsy, and other disease. In patients with two or more underlying diseases treated by steroid administration, each disease was counted in the analyses. Information on the dosage and duration of steroid administration was not obtained.

In order to evaluate the potential bias of hospitals newly participating in the monitoring system, we conducted an additional analysis by limiting the data to that collected by the 11 hospitals that regularly reported patients throughout the study period (Asahikawa Medical University Hospital, Kanazawa Medical University Hospital, Kyoto Prefecture University Hospital, Kyushu University Hospital, Kurume University Hospital, Nagasaki University Hospital, Nagoya University Hospital, Osaka University Hospital, Saga University Hospital, Shinshu University Hospital, and Showa University Fujigaoka Hospital).

Trends in gender, age, potential causative factors, and underlying diseases treated by steroid administration were examined using the Cochran-Armitage test. Statistical tests were employed with the significance level set at 0.05. All *P* values were two-sided. All analyses were performed using SAS version 9.3 (SAS Institute, Cary, NC, USA).

## RESULTS

**Figure 1.**
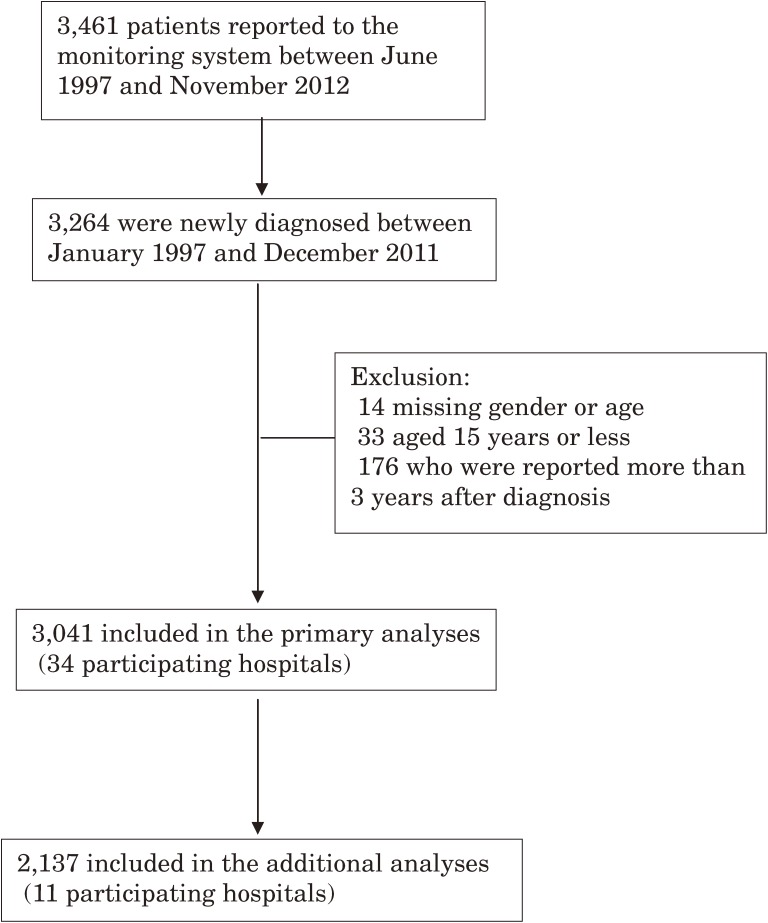
Flow diagram of the selection of subjects for analyses of trends in nontraumatic osteonecrosis of the femoral head. The additional analyses included 11 hospitals that regularly reported patients throughout the study period.

Figure 1 shows the flow diagram of the present study. A total of 3264 patients were diagnosed between January 1997 and December 2011 in 34 participating hospitals. Ultimately, 3041 patients were subjected to the primary analyses, and 2137 patients who were reported by the 11 hospitals were included in additional analyses.

**Figure 2.**
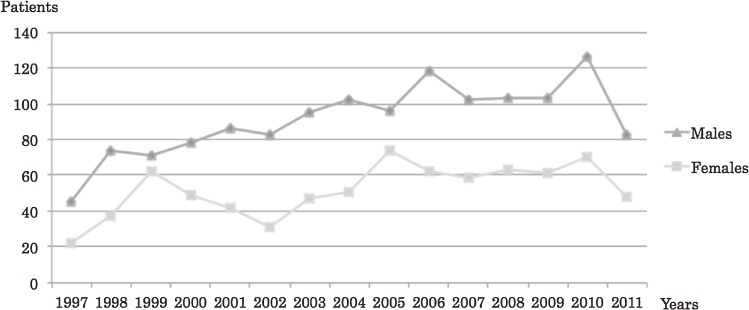
Annual trends in the reported number of newly diagnosed patients with nontraumatic osteonecrosis of the femoral head from 1997–2011 according to gender. The trends are presented for the 11 hospitals which regularly reported patients throughout the study period. The number of patients gradually increased for both sexes until 2006.

The annual number of patients diagnosed at the 11 participating hospitals increased substantially for both genders throughout the study period (Figure 2). However, the increase became fairly subtle after 2006. The number of patients appeared to decline in 2011 because the present analysis did not include those patients who had been diagnosed before 31st December 2011 but had not been reported by November 2012.

The gender ratio (male:female) was 1.7 over the entire period and remained consistent throughout the study period (Table 1). In males, the proportion of patients aged 40–49 years significantly decreased. The proportions of patients aged 50–59, 60–69, and ≥70 years increased, although the differences were not significant. In females, a significant decrease in the proportion of patients aged 16–29 years and significant increases in those aged 30–39 and 60–69 years were observed. Regarding assessment of the distribution of potential causative factors in males, the proportion of patients with habitual alcohol intake was the highest (48%), followed by systemic steroid administration (35%), neither factor (9.6%), and both factors (7.9%). In females, those with systemic steroid administration accounted for the majority of the causes (70%), while a higher proportion of females than males reported neither cause (17%). The average age of males with a history of habitual alcohol intake was 45 (standard deviation [SD], 12) years. Regarding patients treated by steroid administration, the average (SD) age of SLE and non-SLE males was 37 (13) years and 44 (14) years, respectively. In females, the average (SD) age of patients with a history of habitual alcohol intake was 42 (12) years. The average (SD) age of SLE and non-SLE females with a history of steroid administration was 37 (13) years and 49 (14) years, respectively.

**Table 1.** Trends in the distribution of demographic data and potential causative factors according to gender from 1997–2011

	Study period^a^				*P*^b^

	Entire period *n* = 3041	First period *n* = 678	Second period *n* = 1039	Third period *n* = 1324	
Number of participating hospitals	33	16	21	30	
Gender ratio (male:female)	1.7	1.6	1.7	1.6	0.963

*Males*	*(n = 1902)*	*(n = 418)*	*(n = 661)*	*(n = 823)*	
Age (years)					
16–29	250 (13)	63 (15)	91 (14)	96 (12)	0.078
30–39	510 (27)	105 (25)	188 (28)	217 (26)	0.821
40–49	489 (26)	120 (29)	175 (26)	194 (24)	0.043
50–59	385 (20)	80 (19)	120 (18)	185 (22)	0.092
60–69	194 (10)	37 (8.9)	62 (9.4)	95 (12)	0.105
≥70	74 (3.9)	13 (3.1)	25 (3.8)	36 (4.4)	0.270
Potential causative factors					
Systemic steroid administration	660 (35)	152 (36)	237 (36)	271 (33)	0.184
Habitual alcohol intake	906 (48)	193 (46)	319 (48)	394 (48)	0.631
Both	150 (7.9)	32 (7.7)	43 (6.5)	75 (9.1)	0.227
Neither	182 (9.6)	41 (9.8)	59 (9.0)	82 (10)	0.821
Unknown	4	0	3	1	

*Females*	*(n = 1139)*	*(n = 260)*	*(n = 378)*	*(n = 501)*	
Age (years)					
16–29	213 (19)	62 (24)	81 (21)	70 (14)	0.001
30–39	236 (21)	40 (15)	74 (20)	122 (24)	0.003
40–49	197 (17)	49 (19)	62 (16)	86 (17)	0.642
50–59	186 (16)	48 (18)	69 (18)	69 (14)	0.062
60–69	175 (15)	32 (12)	55 (15)	88 (18)	0.048
≥70	132 (12)	29 (11)	37 (9.8)	66 (13)	0.288
Potential causative factors					
Systemic steroid administration	789 (70)	184 (71)	263 (70)	342 (69)	0.548
Habitual alcohol intake	124 (11)	22 (8.5)	48 (13)	54 (11)	0.468
Both	31 (2.7)	2 (0.8)	10 (2.7)	19 (3.8)	0.015
Neither	187 (17)	51 (20)	55 (15)	81 (16)	0.352
Unknown	8	1	2	5	

Values are expressed as numbers (%).

^a^Study period was divided into first (1997–2001), second (2002–2006), and third (2007–2011) periods.

^b^The Cochran-Armitage test.

With respect to alcohol consumption in patients who were reported to the monitoring system after 2009, the average (SD) alcohol intake per day was 74 (55) g of ethanol and the average drinking period was 20 (12) years. All the patients who were reported after 2009 had a history of alcohol consumption of three days a week or more.

Table 2 and Table 3 show underlying diseases treated by systemic steroid administration. In males (Table 2), the most frequent disease was SLE (9.0%), followed by nephrotic syndrome (8.1%), hematological malignancy (8.1%), and bronchial asthma (7.5%). In females (Table 3), SLE was again the most frequent underlying disease treated by steroids (34%), followed by bronchial asthma (6.1%), polymyositis/dermatomyositis (5.7%), and thrombocytopenic purpura (5.5%). The proportion with SLE declined in the third period in both males and females (*P* = 0.077 and *P* = 0.022, respectively). The proportion of patients receiving steroid treatment for renal transplantation fell consistently across the study period in both males and females (*P* = 0.047 and *P* = 0.038, respectively). In contrast, the proportion of patients with pulmonary disease (except asthma) showed consistent increases in both males and females (*P* = 0.022 and *P* = 0.027, respectively). In females, an increase in the proportion of patients receiving steroids for skin disease was observed during the third period (*P* = 0.046).

**Table 2.** Trends in the distribution of underlying diseases for which patients received steroid therapy during 1997–2011 in males

	Study period^a^				*P*^b^

	Entire period *n* = 810	First period *n* = 184	Second period *n* = 280	Third period *n* = 346	
Systemic lupus erythematosus	73 (9.0)	19 (10)	32 (11)	22 (6.4)	0.077
Rheumatoid arthritis	13 (1.6)	5 (2.7)	5 (1.8)	3 (0.9)	0.103
Polymyositis/dermatomyositis	31 (3.8)	6 (3.3)	13 (4.6)	12 (3.5)	0.991
Mixed connective tissue disease	7 (0.9)	3 (1.6)	1 (0.4)	3 (0.9)	0.506
Sjögren syndrome	6 (0.7)	1 (0.5)	2 (0.7)	3 (0.9)	0.663
Other type of collagen disease	45 (5.6)	6 (3.3)	10 (3.5)	29 (8.4)	0.005
Nephrotic syndrome	66 (8.1)	15 (8.2)	23 (8.2)	28 (8.1)	0.984
Nephritis	39 (4.8)	6 (3.3)	16 (5.7)	17 (4.9)	0.475
Renal transplantation	16 (2.0)	7 (3.8)	5 (1.8)	4 (1.2)	0.047
Other organ transplantation^c^	4 (0.5)	0 (0)	2 (0.7)	2 (0.6)	0.433
Hematological malignancy	66 (8.1)	11 (6.0)	25 (8.9)	30 (8.7)	0.313
Thrombocytopenic purpura	33 (4.1)	10 (5.4)	10 (3.6)	13 (4.8)	0.434
Aplastic anemia	12 (1.5)	2 (1.1)	3 (1.1)	7 (2.0)	0.321
Inflammatory bowel disease	50 (6.7)	12 (6.5)	21 (7.5)	17 (4.9)	0.373
Hepatitis	13 (1.6)	2 (1.1)	6 (2.1)	5 (1.4)	0.868
Bronchial asthma	61 (7.5)	11 (6.0)	20 (7.1)	30 (8.6)	0.225
Pulmonary disease^d^	26 (3.2)	1 (0.5)	10 (3.6)	15 (5.5)	0.022
Skin disease	38 (4.7)	9 (4.9)	8 (2.9)	21 (6.1)	0.328
Eye disease	39 (4.8)	8 (4.3)	15 (5.4)	16 (4.6)	0.936
Ear disease	37 (4.6)	11 (6.0)	11 (3.9)	15 (4.3)	0.487
Facial palsy	11 (1.4)	2 (1.1)	4 (1.4)	5 (1.4)	0.739
Other disease	151 (19)	38 (21)	50 (18)	63 (18)	0.606
Unknown	6	1	1	4	

Values are expressed as numbers (%).

^a^Study period was divided into first (1997–2001), second (2002–2006), and third (2007–2011) periods.

^b^The Cochran-Armitage test.

^c^Except renal transplantation and bone marrow transplantation.

^d^Except asthma.

**Table 3.** Trends in the distribution of underlying diseases for which patients received steroid therapy during 1997–2011 in females

	Study period^a^				*P*^b^

	Entire period *n* = 820	First period *n* = 186	Second period *n* = 273	Third period *n* = 361	
Systemic lupus erythematosus	275 (34)	69 (37)	102 (37)	104 (29)	0.022
Rheumatoid arthritis	11 (1.3)	1 (0.5)	3 (1.1)	7 (1.9)	0.160
Polymyositis/dermatomyositis	47 (5.7)	11 (6.0)	14 (5.1)	22 (6.1)	0.870
Mixed connective tissue disease	31 (3.8)	4 (2.2)	13 (4.8)	14 (3.9)	0.395
Sjögren syndrome	23 (2.8)	5 (2.7)	5 (1.8)	13 (3.6)	0.413
Other type of collagen disease	48 (5.9)	4 (2.2)	10 (3.7)	34 (9.4)	<0.001
Nephrotic syndrome	34 (4.1)	7 (3.8)	11 (4.0)	16 (4.4)	0.711
Nephritis	29 (3.5)	4 (2.2)	13 (4.8)	12 (3.3)	0.675
Renal transplantation	15 (1.8)	6 (3.2)	6 (2.2)	3 (0.8)	0.038
Other organ transplantation^c^	4 (0.5)	1 (0.5)	1 (0.4)	2 (0.6)	0.931
Hematological malignancy	39 (4.8)	10 (5.4)	18 (6.6)	11 (3.0)	0.120
Thrombocytopenic purpura	45 (5.5)	14 (7.5)	13 (4.8)	18 (5.0)	0.263
Aplastic anemia	8 (1.0)	2 (1.1)	1 (0.4)	5 (1.4)	0.556
Inflammatory bowel disease	20 (2.4)	8 (4.3)	5 (1.8)	7 (1.9)	0.124
Hepatitis	16 (2.0)	2 (1.1)	4 (1.5)	10 (2.8)	0.143
Bronchial asthma	50 (6.1)	10 (5.4)	17 (6.2)	23 (6.4)	0.682
Pulmonary disease^d^	20 (2.4)	1 (0.5)	6 (2.2)	13 (3.6)	0.027
Skin disease	22 (2.7)	4 (2.2)	2 (0.7)	16 (4.4)	0.046
Eye disease	21 (2.6)	5 (2.7)	6 (2.2)	10 (2.8)	0.896
Ear disease	15 (1.8)	2 (1.1)	5 (1.8)	8 (2.2)	0.389
Facial palsy	8 (1.0)	1 (0.5)	5 (1.8)	2 (0.6)	0.742
Other disease	86 (10)	16 (8.6)	28 (10)	42 (12)	0.280
Unknown	3	0	0	3	

Values are expressed as numbers (%).

^a^Study period was divided into first (1997–2001), second (2002–2006), and third (2007–2011) periods.

^b^The Cochran-Armitage test.

^c^Except renal transplantation and bone marrow transplantation.

^d^Except asthma.

In the additional analysis, which included data from 11 hospitals (eTable 1), the results were unchanged in comparison to the primary analyses except for the following: the change in the proportion of potential causative factors in males was statistically significant, with a decrease from 36% to 27% in systemic steroid administration (*P* = 0.002) and an increase from 47% to 53% in habitual alcohol intake (*P* = 0.104) (eTable 1); regarding underlying diseases treated by steroid administration in males, the proportion of patients treated for RA significantly decreased from 3.2% to 0% (*P* = 0.026), while decreases in the proportion of patients treated for SLE and renal transplantation did not reach statistical significance (eTable 2 and eTable 3).

## DISCUSSION

This study, which analyzed the data of over 3000 patients who were newly diagnosed with ONFH during a 15-year period in Japan, is the first to investigate temporal trends in the characteristics of ONFH. The strengths of our monitoring system include the strict diagnostic criteria, in which all diagnoses were carried out by hip surgeons who were members of the research committee. It has been shown that, in Japan, 12% of patients who had been diagnosed with ONFH and had been reported to the medical claim system were misdiagnosed because some physicians were unfamiliar with ONFH.^11^ Using standardized and consistent criteria over the study period also enhanced the reliability of observed trends. In addition, the monitoring system has many advantages with respect to costs and labor in comparison to a nationwide epidemiologic survey involving random sampling of participating hospitals.^5^

We observed an increase in the annual number of newly-diagnosed patients among the 11 participating hospitals from 1997 to 2006. This finding is consistent with previous reports^3^^–^^5^ and may be partly explained by the spread of the use of magnetic resonance imaging (MRI), which has contributed to accurate diagnosis of ONFH. Organization for Economic Co-operation and Development (OECD) health data showed that the availability of MRI units has increased in most OECD countries over the past two decades.^12^ In Japan, the number of MRI units per million people showed a sharp increase from 1996 to 2005 (23.2 units and 40.1 units, respectively), whereas the increase levelled off in 2008 (43.1 units). Our study also suggests no further increase in the number of patients after 2006.

We investigated trends in patient characteristics, both in all participating hospitals and in the subset of 11 hospitals, to assess potential biases that may have been introduced by newly participating hospitals. Because we did not observe a substantial difference between the two datasets, the results of the primary analyses are discussed below.

The age at disease diagnosis shifted from a younger age toward an older age across the study period, especially in females. One possible reason is the decreased proportion of patients receiving steroid treatment for SLE, which was the most frequent underlying disease treated by steroid administration in the present study and is likely to affect the younger population because the average age of patients treated for SLE was younger than that of non-SLE patients. Another possible reason is the aging population in Japan. The proportion of the population aged 16–29 years among those aged 16 years or older decreased from 23% in 2000 to 17% in 2010, while the proportions of the population aged 30–39 and 60–69 years increased from 13% to 14% and 11% to 13%, respectively.^13^

The proportion of patients with a history of habitual alcohol intake did not show apparent trends. Additional information of patients with ONFH who were reported after 2009 showed that all patients who had a history of alcohol consumption did so on three days a week or more. According to the National Health and Nutrition Survey in Japan, the prevalence of those who drink three days a week or more has been decreasing: in males, values were 53% in 1999, 38% in 2004, and 36% in 2009; in females, rates were 8.1% in 1999, 7.1% in 2004, and 6.9% in 2009.^14^

The proportion of patients with a history of systemic steroid administration did not change during the study period. With respect to individual underlying diseases treated by steroid administration, proportions of patients treated for SLE and renal transplantation decreased in both genders. In Japan, SLE patients receiving public financial aid for treatment have been increasing: there were 47 295 patients in 1999, 52 195 in 2004, and 57 253 in 2009.^15^ Also, an increase in number of patients with renal transplantation was shown: 158 patients in 1999, 173 in 2004, and 182 in 2009.^16^ However, current treatments for SLE and prevention of rejection after renal transplantation have improved and provided steroid-sparing options,^17^^–^^19^ which are expected to contribute to the significant reduction in the number of steroid-induced ONFH cases. In contrast, the number of patients with pulmonary diseases other than asthma significantly increased. The majority of cases were chronic obstructive pulmonary disorder (COPD) and interstitial pneumonia (IP). These findings are consistent with the increasing trends in COPD and IP documented in Japan.^15^^,^^20^ Most patients admitted with exacerbation of COPD are likely to be treated with high-dose steroids, although a study has shown that low doses of steroids are not associated with worse outcomes.^21^ Because the treatment for IP has been changing towards a steroid-sparing regimen,^22^ a decline in ONFH may be observed in the future.

The shift in age distribution in this study population might also influence trends in underlying diseases which needed steroid administration, especially in females. The average ages of females with underlying diseases treated with steroids that showed a significant increase were relatively old: 56 years in other types of collagen disease; 58 years in pulmonary disease; and 53 years in skin disease. In contrast, the average age of females with underlying diseases that showed a significant decline was relatively young: 37 years in SLE and 37 years in renal transplantation. A similar relationship was observed in males.

There are several limitations to this study. First, our findings might be biased because the monitoring system is based solely on data from university hospitals and highly specialized hospitals. However, when we compared our findings with the results of a 2004 nationwide epidemiologic survey in Japan,^5^ we did not find any substantial differences between the two studies. The nationwide survey showed a similar distribution of age at diagnosis to this study and a gender ratio of 1.5, which was slightly lower than in the present study. In addition, assessment of potential causative factors in that study found 34% of patients with systemic steroid administration, 47% with habitual alcohol intake, 4% with both factors, and 15% with neither factor among males; in females, 76% of patients had systemic steroid administration, 6% had habitual alcohol intake, 1% had both factors, and 16% had neither. Because ONFH is designated as an intractable disease in Japan, patients are likely to visit or be referred to highly specialized hospitals, which might mean that similar characteristics are reported in both the monitoring system and the nationwide survey. Second, although we collected data on the stage and type of ONFH, we could not assess trends in these characteristics due to the revision of their definitions in 2001. Third, a significant increase in the proportion of patients with skin disease as an underlying disease treated by steroid administration in females was demonstrated, but the details of the disease entity were unknown.

### Conclusions

This study confirms that the monitoring system is a useful method for documenting temporal trends in the characteristics of ONFH. Main findings include an increase in the number of patients, a shift to an older age at diagnosis, a decline in the proportion of patients with SLE and renal transplantation, and an increase in the proportion of patients with pulmonary disease (except asthma) as underlying diseases treated by systemic steroid administration. Because our study was a descriptive epidemiologic study, further analytical studies are needed to elucidate the factors associated with these trends.

## ONLINE ONLY MATERIALS

eTable 1. Trends in the distribution of demographic data and assessment of potential causative factors according to gender in the 11 hospitals between 1997 and 2011.

eTable 2. Trends in the distribution of underlying diseases for which patients received steroid therapy in the 11 hospitals between 1997 and 2011 in males.

eTable 3. Trends in the distribution of underlying diseases for which patients received steroid therapy in the 11 hospitals between 1997 and 2011 in females.

## APPENDIX

Members of the Study Group (shown in alphabetical order of affiliation) are as follows: Drs. Takeo Matsuno and Hiroshi Ito (Asahikawa Medical University); Drs. Shunji Kishida and Junichi Nakamura (Chiba University); Drs. Yoshihide Nakamura and Masaki Kishiya (Hirosaki University); Drs. Yuji Yasunaga and Takuma Yamasaki (Hiroshima University); Drs. Daisuke Takahashi, Tsuyoshi Asano and Tokifumi Majima (Hokkaido University); Drs. Setsuro Komiya, Yasuhiro Ishidou and Yoshiya Arishima (Kagoshima University); Dr. Tamon Kabata (Kanazawa University); Drs. Tadami Matsumoto and Ayumi Kaneuji (Kanazawa Medical University); Dr. Kenji Ohzono (Kansai Rosai Hospital); Dr. Moritoshi Itoman (Kitasato University); Drs. Takayuki Nishiyama and Takaaki Fujishiro (Kobe University); Drs. Fujio Higuchi and Takahiro Okawa (Kurume University Medical Center); Drs. Mikihiro Fujioka and Keiichiro Ueshima (Kyoto Prefectural University of Medicine); Dr. Goro Motomura (Kyushu University); Dr. Akihiro Sudo (Mie University); Dr. Etsuo Chosa (Miyazaki University); Dr. Makoto Osaki (Nagasaki University); Dr. Yukiharu Hasegawa (Nagoya University); Drs. Naoto Endo and Kunihiko Tokunaga (Niigata University); Dr. Nobuhiro Kaku (Oita University); Drs. Takashi Nishii, Takashi Sakai and Masaki Takao (Osaka University); Drs. Kunio Takaoka, Hiroaki Nakamura and Hiroyoshi Iwaki (Osaka City University); Dr. Hidenobu Miki (Osaka National Hospital); Drs. Takao Hotokebuchi and Masaaki Mawatari (Saga University); Drs. Setsuo Ninomiya and Hitoshi Taneda (Saitama Medical School); Dr. Satoshi Nagoya (Sapporo Medical University); Dr. Hiroyuki Kodaira (Shinshu University School of Medicine); Dr. Takashi Atsumi (Showa university Fujigaoka hospital); Dr. Seneki Kobayashi (Suwa Red Cross Hospital); Drs. Sakae Tanaka and Hideya Ito (The University of Tokyo); Dr. Kengo Yamamoto (Tokyo Medical University); Drs. Tetsuya Jinno and Daisuke Koga (Tokyo Medical and Dental University); Drs. Michiaki Takagi and Kan Sasaki (Yamagata University); and Drs. Yutaka Inaba and Naomi Kobayashi (Yokohama City University).
