# Supplementary material for: Temporal Trends in Characteristics of Newly Diagnosed Nontraumatic Osteonecrosis of the Femoral Head From 1997 to 2011: A Hospital-Based Sentinel Monitoring System in Japan
Source: J Epidemiol. 2015 Jun 5;25(6):437–44. doi: 10.2188/jea.JE20140162 (PMC4444498; doi:10.2188/jea.JE20140162)
Supplement: eTable 1. [file je-25-437-s001.pdf]

eTable 1. Trends in the distribution of demographic data and potential causative factors according to gender in the 11 hospitals between 1997 and 2011

	Study period[a]				
	Entire period n=2,137	First period n=564	Second period n=755	Third period n=818	*P*[b]
Gender ratio (male:female)	1.7	1.7	1.8	1.7	0.844
Males	*(n=1,359)*	*(n=352)*	*(n=490)*	*(n=517)*	
Age (years)					
16-29	176 (13)	50 (14)	71 (14)	55 (11)	0.137
30-39	385 (28)	91 (26)	143 (29)	151 (29)	0.313
40-49	346 (25)	100 (28)	132 (27)	114 (22)	0.027
50-59	272 (20)	67 (19)	88 (18)	117 (23)	0.145
60-69	134 (9.9)	33 (9.4)	40 (8.2)	61 (12)	0.177
≥70	36 (3.4)	11 (3.1)	16 (3.3)	19 (3.7)	0.647
Potential causative factors					
Systemic steroid administration	429 (32)	128 (36)	163 (33)	138 (27)	0.002
Habitual alcohol intake	689 (51)	166 (47)	250 (51)	273 (53)	0.104
Both	109 (8.0)	29 (8.2)	30 (6.2)	50 (9.7)	0.324
Neither	129 (9.5)	29 (8.2)	45 (9.2)	55 (11)	0.224
Unknown	3	0	2	1	
Females	*(n=778)*	*(n=212)*	*(n=265)*	*(n=301)*	
Age (years)					
16-29	155 (20)	53 (25)	60 (23)	42 (14)	0.001
30-39	153 (20)	29 (14)	48 (18)	76 (25)	0.001
40-49	142 (18)	40 (19)	47 (18)	55 (18)	0.886
50-59	134 (17)	38 (18)	53 (20)	43 (14)	0.223
60-69	109 (14)	26 (12)	36 (14)	47 (16)	0.273
≥70	85 (11)	26 (12)	21 (7.9)	38 (13)	0.745
Potential causative factors					
Systemic steroid administration	534 (69)	153 (73)	182 (69)	199 (67)	0.169
Habitual alcohol intake	79 (10)	17 (8.1)	31 (12)	31 (10)	0.452

Both	20 (2.6)	1 (0.5)	7 (2.7)	12 (4.0)	0.014
Neither	139 (18)	40 (19)	43 (16)	56 (19)	0.969
Unknown	6	1	2	3	

Values are expressed as numbers (%).

[a] Study period was divided into first (1997-2001), second (2002-2006), and third (2007-2011) periods.

[b] the Cochran-Armitage test
