# Supplementary material for: Temporal Trends in Characteristics of Newly Diagnosed Nontraumatic Osteonecrosis of the Femoral Head From 1997 to 2011: A Hospital-Based Sentinel Monitoring System in Japan
Source: J Epidemiol. 2015 Jun 5;25(6):437–44. doi: 10.2188/jea.JE20140162 (PMC4444498; doi:10.2188/jea.JE20140162)
Supplement: eTable 2. [file je-25-437-s002.pdf]

**eTable 2.** Trends in the distribution of underlying diseases for which patients received steroid therapy in the 11 hospitals between 1997 and 2011 in males

| | Study period[a] | | | | |
|---|---|---|---|---|---|
| | Entire period n=538 | First period n=157 | Second period n=193 | Third period n=188 | *P*[b] |
| Systemic lupus erythematosus | 46 (8.6) | 15 (9.6) | 18 (9.3) | 13 (6.9) | 0.389 |
| Rheumatoid arthritis | 10 (1.9) | 5 (3.2) | 5 (2.6) | 0 (0) | 0.026 |
| Polymyositis/dermatomyositis | 15 (2.8) | 5 (3.2) | 6 (3.1) | 4 (2.1) | 0.554 |
| Mixed connective tissue disease | 3 (0.6) | 2 (1.3) | 0 (0) | 1 (0.5) | 0.398 |
| Sjögren syndrome | 6 (1.1) | 1 (0.6) | 2 (1.0) | 3 (1.6) | 0.385 |
| Other type of collagen disease | 22 (4.1) | 5 (3.2) | 6 (3.1) | 11 (5.9) | 0.185 |
| Nephrotic syndrome | 48 (8.9) | 12 (7.6) | 20 (10) | 16 (8.5) | 0.782 |
| Nephritis | 25 (4.6) | 5 (3.2) | 11 (5.7) | 9 (4.8) | 0.491 |
| Renal transplantation | 15 (2.8) | 7 (4.5) | 4 (2.1) | 4 (2.1) | 0.211 |
| Other organ transplantation[c] | 3 (0.6) | 0 (0) | 2 (1.0) | 1 (0.5) | 0.540 |
| Hematological malignancy | 47 (8.7) | 11 (7.0) | 18 (9.3) | 18 (9.6) | 0.387 |
| Thrombocytopenic purpura | 23 (5.6) | 9 (5.7) | 7 (3.6) | 7 (3.7) | 0.387 |
| Aplastic anemia | 7 (1.3) | 1 (0.6) | 3 (1.6) | 3 (1.6) | 0.435 |
| Inflammatory bowel disease | 33 (6.1) | 10 (6.4) | 13 (6.7) | 10 (5.3) | 0.692 |
| Hepatitis | 9 (1.7) | 2 (1.3) | 5 (2.6) | 2 (1.1) | 0.840 |
| Bronchial asthma | 37 (6.9) | 10 (6.4) | 11 (5.7) | 16 (8.5) | 0.387 |
| Pulmonary disease[d] | 14 (2.6) | 1 (0.6) | 6 (3.1) | 7 (3.7) | 0.073 |
| Skin disease | 26 (4.8) | 9 (5.7) | 6 (3.1) | 11 (5.9) | 0.876 |
| Eye disease | 25 (4.6) | 7 (4.5) | 11 (5.7) | 7 (3.7) | 0.732 |
| Ear disease | 31 (5.8) | 9 (5.7) | 8 (4.1) | 14 (7.4) | 0.435 |
| Facial palsy | 6 (1.1) | 2 (1.3) | 1 (0.5) | 3 (1.6) | 0.725 |
| Other disease | 100 (19) | 31 (20) | 40 (21) | 29 (15) | 0.308 |
| Unknown | 5 | 1 | 0 | 4 | |

Values are expressed as numbers (%).

[a] Study period was divided into first (1997-2001), second (2002-2006), and third (2007-2011) periods.

[b] the Cochran-Armitage test

[c] Except renal transplantation and bone marrow transplantation

[d] Except asthma
