# Supplementary material for: Temporal Trends in Characteristics of Newly Diagnosed Nontraumatic Osteonecrosis of the Femoral Head From 1997 to 2011: A Hospital-Based Sentinel Monitoring System in Japan
Source: J Epidemiol. 2015 Jun 5;25(6):437–44. doi: 10.2188/jea.JE20140162 (PMC4444498; doi:10.2188/jea.JE20140162)
Supplement: eTable 3. [file je-25-437-s003.pdf]

eTable 3. Trends in the distribution of underlying diseases for which patients received steroid therapy in the 11 hospitals between 1997 and 2011 in females

	Study period[a]				
	Entire period n=554	First period n=154	Second period n=189	Third period n=211	*P*[b]
Systemic lupus erythematosus	179 (32)	58 (38)	69 (37)	52 (25)	0.005
Rheumatoid arthritis	7 (1.3)	1 (0.7)	2 (1.1)	4 (1.9)	0.280
Polymyositis/dermatomyositis	35 (6.3)	8 (5.2)	10 (5.3)	17 (8.1)	0.242
Mixed connective tissue disease	22 (4.0)	3 (2.0)	9 (4.8)	10 (4.7)	0.200
Sjögren syndrome	10 (1.8)	3 (2.0)	4 (2.1)	3 (1.4)	0.679
Other type of collagen disease	21 (3.8)	4 (2.6)	3 (1.6)	14 (6.6)	0.030
Nephrotic syndrome	27 (4.9)	6 (3.9)	9 (4.8)	12 (5.7)	0.431
Nephritis	21 (3.8)	4 (2.6)	10 (5.3)	7 (3.3)	0.820
Renal transplantation	13 (2.3)	6 (3.9)	5 (2.6)	2 (0.9)	0.061
Other organ transplantation[c]	3 (0.5)	1 (0.7)	1 (0.5)	1 (0.5)	0.822
Hematological malignancy	33 (6.0)	10 (6.5)	16 (8.5)	7 (3.3)	0.151
Thrombocytopenic purpura	33 (6.0)	12 (7.8)	7 (3.7)	14 (6.6)	0.750
Aplastic anemia	4 (0.7)	1 (0.7)	1 (0.5)	2 (0.9)	0.714
Inflammatory bowel disease	14 (2.5)	6 (3.9)	3 (1.6)	5 (2.4)	0.407
Hepatitis	11 (2.0)	1 (0.7)	4 (2.1)	6 (2.8)	0.142
Bronchial asthma	36 (6.5)	7 (4.6)	13 (6.9)	16 (7.6)	0.246
Pulmonary disease[d]	14 (2.5)	1 (0.7)	6 (3.2)	7 (3.3)	0.086
Skin disease	18 (3.2)	3 (2.0)	2 (1.1)	13 (6.2)	0.015
Eye disease	17 (3.1)	5 (3.3)	5 (2.6)	7 (3.3)	0.942
Ear disease	9 (1.6)	2 (1.3)	2 (1.1)	5 (2.4)	0.386
Facial palsy	7 (1.3)	1 (0.7)	4 (2.1)	2 (0.9)	0.897
Other disease	54 (9.7)	13 (8.5)	16 (8.5)	25 (12)	0.253
Unknown	1	0	0	1	

Values are expressed as numbers (%).

[a] Study period was divided into first (1997-2001), second (2002-2006), and third (2007-2011) periods.

[b] the Cochran-Armitage test

[c] Except renal transplantation and bone marrow transplantation

[d] Except asthma
